# Resilience, disease and the age of single cell science

**DOI:** 10.18632/aging.102850

**Published:** 2020-02-10

**Authors:** Michael Simons, Pei-Yu Chen, Thomas W. Chittenden

**Affiliations:** 1 Yale Cardiovascular Research Center, New Haven, CT 06510, USA; 2 Department of Cell Biology, Yale University School of Medicine, New Haven, CT 06510, USA; 3 WuXi NextCode, Inc., Cambridge, MA 02142, USA; 4 Divison of Genetics and Genomics, Boston Children’s Hospital, Harvard Medical School, Boston, MA 02115, USA

**Keywords:** atherosclerosis, EndMT, aging, inflammation, artificial intelligence, endothelial-to-mesenchymal transition

Recent evidence has placed endothelial-to-mesenchymal transition (EndMT) at the center of numerous pathological conditions from atherosclerosis to pulmonary hypertension, renal dysfunction and vascular malformations among others [1,2]. Importantly, similar changes have been observed in the aging vasculature and may play an important role in the aging process itself [3]. The primary trigger of EndMT is chronic vascular inflammation that leads to activation of endothelial TGFβ signaling that, in turns, promotes activation of the EndMT transcriptional program. Once triggered, EndMT becomes self-sustaining, gradually driving disease (or aging) forward even if the initial trigger has been removed [4].

The first proof of this hypothesis came in the study of Chen et al. that used atherosclerosis as a model. Prior studies have demonstrated a frequent occurrence of EndMT in mouse and human atherosclerotic plaques [1,5]. Furthermore, an earlier study from the Simons laboratory demonstrated acceleration of atherosclerosis following increase in EndMT [5]. In the current study, Chen and colleagues using genetic techniques to induce endothelial-specific deletion of TGFβ receptor 1 and 2 genes in adult fate-mapped ApoE^-/-^ mice either at the time of introduction of high-fat diet or after three months of such diet when atherosclerotic plaques were fully formed. Thus induced, inhibition of TGFβ signaling not only arrested disease progression but resulted in a dramatic regression of fully established, mature, atherosclerotic plaques [6]. The authors then went on to demonstrate that nanoparticle-based delivery of RNAi targeting TGFβ receptors achieves the same therapeutic benefit. These observation raises hope that inhibition of EndMT can arrest progression of certain diseases and, clearly, the effect of this intervention on aging deserves close scrutiny.

Another important aspect of this study was a single cell RNA-seq (scRNA-seq)-based analysis of gene expression in various subsets of normal and diseased endothelial cells. While the recent advent of scRNA-seq is rapidly transforming our understanding of normal biology and disease pathogenesis, the tool has not yet lived up to its full potential. In part this is due to technical limitations of the current technology. A larger challenge is the analysis of vast amounts of the sequencing data. Here the introduction of artificial intelligence (AI) and statistical machine learning techniques hold significant potential for robustly addressing current unresolved issues in large-scale statistical optimization of high-dimensional, genome-wide scRNA-seq data. Of particular interest, unsupervised generative AI models, such as variational autoencoders (VAEs) are capable of robust dimensionality reduction and subsequently learned reconstruction of the original input or latent feature space. Without dependence upon *a priori* class annotation, these deep artificial neural network topologies hold great promise for uncovering the dysregulated molecular signals that drive aberrant cellular behaviour in an unbiased manner. By fitting a novel zero-inflated negative binomial VAE to probabilistically control for technical dropout of scRNA-seq data, Chen et al. were able to identify a specific subset of endothelial cells that appeared to be the primary driver of EndMT and, hence, atherosclerotic plaque growth. Importantly, the size of this pathogenic cell population was decisively reduced by inhibition of endothelial TGFβ signaling. Whether these cells are truly principal drivers of disease what the origin of this population is and how different they are from other cell populations will require longitudinal analysis of scRNA-seq data and applications of new AI tools to their analysis.

These observations fit with the general framework of extensive cellular heterogeneity under both normal and disease [7,8]. Importantly, molecular basis and functional significance of this heterogeneity are unknown. One could hypothesize some endothelial subsets are more pathogenic than others while others are disease resistant. Identifying and characterizing such disease-prone vs. resistant using a variety of single cell analysis approaches could be crucial to the understanding of molecular basis of vascular disease pathogenesis, resilience and aging.
